# Exploring the Effects of MXene on Nonisothermal Crystallization and Melting Behavior of β-Nucleated Isotactic Polypropylene

**DOI:** 10.3390/polym13213815

**Published:** 2021-11-04

**Authors:** Wanxin Peng, Furui Sun, Yuke Liang, Jian Kang, Jinyao Chen, Wei Wang, Ya Cao, Ming Xiang

**Affiliations:** 1 State Key Laboratory of Polymer Materials Engineering, Polymer Research Institute of Sichuan University, Chengdu 610065, China; kelly_520_peng@163.com (W.P.); 2020223090075@stu.scu.edu.cn (Y.L.); jiankang@scu.edu.cn (J.K.); caoya@scu.edu.cn (Y.C.); xiangming@scu.edu.cn (M.X.); 2 Science and Technology on Advanced Functional Composites Laboratory, Aerospace Research Institute of Materials and Processing Technology, Beijing 100076, China; sunfurui1202@163.com

**Keywords:** istotactic polypropylene, MXene, nonisothermal crystallization behavior

## Abstract

In this study, one of the commonly used MXene (Ti_3_C_2_T_x_) and β nucleated isotactic polypropylene (β-iPP)/MXene composites of different compositions were fabricated. The effects of MXene on non-isothermal crystallization and polymorphic behavior of β-iPP/MXene composites were comparatively studied. The non-isothermal crystallization kinetics indicates that for all samples, the lower cooling rates promote composites to crystallize at higher temperatures. When MXene and β-Nucleating agent (β-NA) are added separately, the crystallization temperature of composites shifts towards higher temperatures at all cooling rates. When MXene and β-NA are added simultaneously, the composite shows different cooling rate dependence, and the effects of improving crystallization temperatures is more obvious under rapid cooling. The activation energy of four samples iPP, iPP/MXene, iPP/β-NA, and iPP/MXene/β-NA were −167.5, −185.5, −233.8, and −218.1 kJ/mol respectively, which agree with the variation tendency of crystallization temperatures. The polymorphic behavior analysis obtained from Differential Scanning calorimetry (DSC) is affected by two factors: the ability to form β-crystals and the thermal stability of β-crystals. Because β-crystals tend to recrystallize to α-crystals below a critical temperature, to eliminate the effect of β-α recrystallization, the melting curves at end temperatures T_end_ = 50 °C and T_end_ = 100 °C are comparatively studied. The results show that more thermally unstable β-crystals would participate in β-α recrystallization with higher cooling rates. Moreover, thermal stability of β-crystals is improved by adding MXene. To further verify these findings, samples of three different thermal conditions were synthesized and analyzed by DSC, X-Ray Diffraction (XRD), and Polarized Light Optical Microscopy (PLOM), and the results were consistent with the above findings. New understandings of synthesizing β-iPP/MXene composites with adjustable morphologies and polymorphic behavior were proposed.

## 1. Introduction

Isotactic polypropylene (iPP), firstly synthesized by Natta et al. in the laboratory and industrialized in 1957 [1], has become one of the most widely applied thermoplastic polymers benefiting from its balance between excellent mechanical properties, versatile processability, and low manufacturing cost [2,3]. iPP is a semicrystalline polymer and shows interesting polymorphic behaviors [4] including monoclinic α-crystal, trigonal β-crystal, triclinic γ-crystal, and smectic forms exhibiting different properties [3,5]. α-crystal is the most stable and is commonly found in iPP, it can be obtained by practical processing conditions [6]. β-crystal is thermodynamically metastable and can only be formed under particular conditions such as crystallization in temperature gradient field [7,8], melt shear field [9], or in the presence of β-nucleating agent (β-NA) [2,10]. Adding β-NA is the most effective way to form high content of β-crystals, which can improve toughness but decreases the stiffness at the same time. On the contrary, adding α-NA promotes the formation of high content α-crystals, which strengthens the material but decreases toughness [11]. As β-crystals have better toughness and α-crystals have better stiffness, the balance between toughness and stiffness can be achieved by efficient control of the polymorphic behavior of iPP broadening its potential applications. Wang et al. [12] worked to compound calcium pimelate as β-NA and multi-wall carbon nanotubes (MWCNTs) as α-NA with iPP to fabricate toughened composites without significant loss in strength and stiffness. In the previous works of our team, it was found that by adjusting the ratio of β-NA and graphene oxide (GO), the ordered structure was favorable for the formation of β-crystals [13]. Moreover, the morphology and crystallization behavior can even be controlled by tuning the fusion temperature and melting time [14].

In the past decade, two-dimensional (2D) materials such as graphene and black phosphorus have drawn significant attention because of their unique structure. They possess superior properties showing potential application in fields such as energy storage, supercapacitor, composites, etc. Recently, Ahmadivand et al. [15] have reported tuning of toroidal resonances and active modulation by gating the graphene monolayer. This finding proved the viability of potential application of graphene in photonics [16]. In 2011, Gogotsi et al. [17] discovered a new type of 2D material, MXenes, which are single layer 2D transitional metal carbides/nitrides. The general formula of MXenes is M_n+1_X_n_T_x_, where M is an early transitional metal element, X is carbon (C) or nitrogen (N) element, T**_x_** represents the various surface terminations such as fluorine, hydroxyl, and/or oxygen atoms [18]. MXenes are commonly obtained by etching from their precursors, MAX phases, which are layered ternary carbides and/or nitrides with a general formula M_n+_1AX_n_ where n generally varies between 1 and 3, and A is an A-group element (mostly groups 13 and 14) such as Al, Si, P, S, Ga, etc. [19,20,21]. MAX phases have layered hexagonal structures, where the A-layers atoms are relatively weakly bonded and can act as potential active sites to remove. The name “MXene” was given due to its similar structure to graphene. In addition, the chemical versatility of MAX phases leads to the chemical diversity of the MXenes family. Currently, about 30 different types of MXenes have been successfully synthesized [18,22]. Among them, Ti_3_C_2_T_x_ is one of the most promising materials and has been widely studied and researched [17,23]. Like other 2D materials, MXenes possess unique properties such as layered structure [24], high electronic conductivity [25,26], large specific area [27], hydrophilicity [28], excellent mechanical properties [29], and have great potential in applications including energy storage, electromagnetic interference (EMI) shielding, water purification, and structural composites. Mathis et al. [26] recently discovered that by adding excessive aluminum during the synthesis of Ti_3_AlC_2_ can lead to superior behavior in electronic conductivity up to 20,000 S/cm, expanding the applications of MXene. Several studies have shown that Ti_3_C_2_T_x_ acts as a potential candidate for fillers in polymer composites. Ling et al. firstly synthesized polyvinyl alcohol (PVA)/ Ti_3_C_2_T_x_ composites with high electrical conductivities and achieved a 300% increase in tensile strength compared with pure PVA films when introducing 40 wt% Ti_3_C_2_T_x_. Yi et al. [30] produced poly(lactic acid) (PLA)/Ti_3_C_2_T_x_ composites and investigated the mechanical properties and crystallization behavior. With the addition of 0.5 wt% Ti_3_C_2_T_x_, the elongation at break was improved 5.9-fold (up to 131.6%), and the crystallinity was also improved due to the heterogeneous nucleation effect. Zhang et al. [31] fabricated ultrahigh molecular weight polyethylene (UHMWPE)/ Ti_3_C_2_T_x_ composite and achieved maximum tensile strength at 0.75 wt% concentration of Ti_3_C_2_T_x_. Wan et al. [32] prepared Ti_3_C_2_T_x_ composite film with balanced shielding performance and mechanical property by introducing poly(3,4-ethylenedioxythiophene)/poly(styrenesulfonate) (PEDOT/PSS) treated in sulfuric acid. The synthesized Ti_3_C_2_T_x_ composite film exhibited EMI SE around 40.5 dB and tensile strength around 38.5 MPa with thickness of 6.6 μm.

While these works focus on improving the physical properties of polymer/MXene composites, the crystallization behavior of composites is still less reported. Huang et al. investigated the crystallization behavior of Poly(ethylene oxide) (PEO)/Ti_3_C_2_T_x_ composites at different concentrations. They found that the crystallization process is promoted below 0.5 wt% Ti_3_C_2_T_x_ due to the heterogeneous nucleation effect, but slows down with further increasing Ti_3_C_2_T_x_ content because of the rigid confinement network [33]. Similar results were also found in our previous work in studying the non-isothermal crystallization behavior of iPP/Ti_3_C_2_T_x_ composites. When 0.5 wt% Ti_3_C_2_T_x_ was added, the peak crystallization temperature and crystallization rate both increased. Once the concentration of Ti_3_C_2_T_x_ reached 1 wt%, the crystallization process was retarded, which might be caused by the confined network [34].

It is well-known that crystallization behaviors can affect the physical and mechanical properties of composites. Moreover, the practical manufacturing process is usually proceeded under non-isothermal crystallization conditions, understanding the non-isothermal crystallization is of great importance. To our best knowledge, the roles of MXene in the polymorphic behavior, crystalline morphologies of β-iPP at varied cooling conditions had not been investigated yet. Thereby, this work chooses the common MXene Ti_3_C_2_T_x_ and fabricates β-iPP/MXene composites to investigate the non-isothermal crystallization kinetics and polymorphic behavior of composites by differential scanning calorimetry (DSC), X-Ray Diffraction (XRD), and polarized light optical microscopy (PLOM). New understandings in preparing the β-iPP/MXene composites with tunable morphologies and polymorphic behavior were also proposed.

## 2. Experimental Section

### 2.1. Materials

The precursor MAX phase Ti_3_AlC_2_ (400 mesh, 99% purity) was purchased from 11 Technology Co. Ltd. (Beijing, China). Lithium fluoride powders (LiF, 99% purity) were purchased from Aladdin Bio-Chem Technology Co. Ltd. (Shanghai, China). Concentrated hydrochloric acid (HCl) with 37 wt% concentration was obtained from Chengdu Kelong Chemical Reagent Factory (Chengdu, China).

The isotactic polypropylene resin (tradename T38F) was obtained from Lanzhou PetroChemical Corp. (Lanzhou, China). The molecular weight was 347,000 gmol^−1^ and the average isotacticity was around 97.6%. The β-nucleating agent (β-NA) used in the work was WBG-II, which is a commercial nucleating gent. The general formula of WBG-II is Ca_x_La_1−x_(LIG1)_m_(LIG2)_n_, where LIG1 and LIG2 are dicarboxylic acid and amide-type ligands [35,36].

### 2.2. Sample Preparation

#### 2.2.1. Etching of MXene

**Figure 1:**
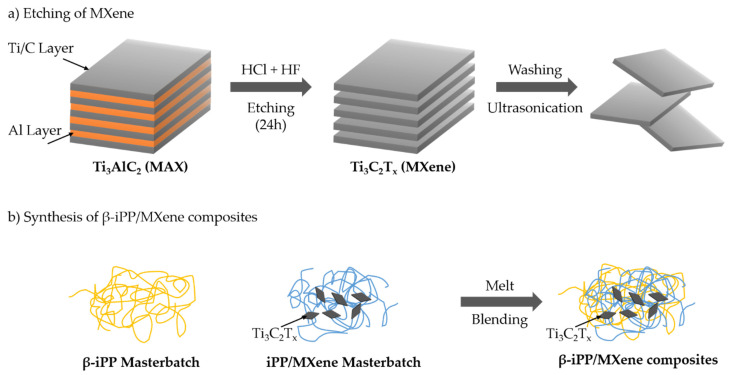
Schematic diagram of etching MXene and synthesis of β-iPP/MXene composites.

To selectively etch the precursor Ti_3_AlC_2_, HCl and LiF were used to in situ form HF. The schematic diagram of etching Ti3AlC2 into Ti3C2Tx is indicated in Figure 1. In the first step, 34 mL HCl was slowly added to 33 mL distilled water. A total of 2 g LiF powder was dissolved in the diluted HCl under magnetic stirring for 10 min. Then, 3 g Ti_3_AlC_2_ was immersed slowly into the solution and kept under magnetic stirring for 24 h at a temperature of 40 °C to allow full reaction. When the etching process was completed, the mixed solution was washed with distilled water and centrifuged at 8000 rpm for 10 min to separate the supernatant from the Ti_3_C_2_T_x_ sediment. This washing–centrifugation cycle was repeated until the pH value of the supernatant reached around 6. The sediment was immersed in distilled water for ultrasonication in the ice bath water for 2 h. The mixture was then centrifuged again to collect the final supernatant and dried under vacuum.

#### 2.2.2. Synthesis of β-iPP/MXene Composites

The composites were prepared by melt blending via a Mini-Lab Extruder (HAAKE MiniLab II, Thermo Fisher Scientific Corp., Waltham, MA, USA). The Ti_3_C_2_T_x_ and β-NA WBG-II were firstly mixed with iPP resin by the Mini-Lab extruder at a temperature of 200 °C and screw speed of 80 rpm to prepare two masterbatches with 2.5 wt% concentration. In the second step, the masterbatches were mixed again with iPP resin to prepare four different samples. The prepared samples were then pressed by a pressure molding machine under 190 °C and 8 MPa for further characterization. To benefit the following discussion, the prepared samples were named as neat iPP, iPP/MXene, iPP/β-NA, and iPP/MXene/β-NA. The concentrations of Ti_3_C_2_T_x_ and WBG-II were fixed at 0.5 wt% and 0.1 wt%, which were chosen based on some previous studies [2,34,37,38]. The schematic diagram of synthesizing the β-iPP/MXene composites is shown in Figure 1.

### 2.3. Characterization

#### 2.3.1. X-ray Diffraction (XRD)

XRD measurements were performed using a diffractor (Ultima IV, Rigaku, Japan) with a *Cu K**_α_*** radiation (*λ* = 0.154 nm) with a voltage of 40 kV and filament current of 40 mA. To measure the spectra of Ti_3_AlC_2_ and Ti_3_C_2_T_x_ powders, the scanning range was set to 2*θ* = 2 − 80° and the scanning rate was 10°/min. Before measuring iPP composites, the samples were firstly hot molded into sheets with 1 mm thickness, and the scanning range was 2*θ* = 5 − 40° at a scanning rate of 2°/min. The relative content of the β phase (k*_β_*) could be calculated from the XRD spectra via the following equation [12,39]:

(1) $$k_{\beta }=\frac{H_{\beta }\left(300\right)}{H_{\beta }\left(300\right)+H_{\alpha }\left(110\right)+H_{\alpha }\left(040\right)+H_{\alpha }\left(130\right)}$$

where *H_β_*(300) denotes the intensity of (300) reflection of *β* phase. *H_α_*(110), *H_α_*(040), and *H_α_*(130) denote intensities of the three strongest reflections of α phase.

#### 2.3.2. Scanning Electron Microscopy (SEM) and Energy Dispersive Spectroscopy (EDS)

The morphology of Ti_3_AlC_2_ and Ti_3_C_2_T_x_ powders were observed by SEM (Apreo S HiVoc, Thermo Fisher Scientific Corp., Waltham, MA, USA) equipped with EDS. The voltage was 5 kV and working distance was 4.9 mm.

#### 2.3.3. Transmission Electron Microscope (TEM)

TEM characterization was conducted on a Tecnai G2 F20 S-TWIN (FEI Corp., Hillsboro, OR, USA) with an accelerating voltage of 200 kV. To observe the structure and dispersion of Ti_3_C_2_T_x_, the sample was dispersed in distilled water under ultrasonication for 10 min. Then, the solution was dropped on a copper grid for observation.

#### 2.3.4. Differential Scanning Calorimetry (DSC)

A Mettler Toledo DSC3 (Mettler Tolado Corp., Zurich, Switzerland) differential scanning calorimetry was used to perform the nonisothermal crystallization experiments under a continuous nitrogen flow of 50 mL min^−1^. For each experiment, the standard procedure was applied as follows: 3–5 mg sample was weighted and heated to 200 °C to erase the previous thermal history. Then, the sample was cooled to end temperature 50 °C at a cooling rate of 5, 10, 20, 30, and 40 °C/min, respectively, and reheated to 200 °C at 10 °C/min to analyze its crystallization and following melting behavior. To ensure the accuracy of the data, the sample was repeatedly tested five to eight times to obtain the average value.

The relative degree of crystallinity (*X_t_*) as a function of temperature (*T*) can be calculated by the following equation [40,41]:

(2) $$X_{t}=\overset{T}{\underset{T_{0}}{\int }}\left(dH/dT\right)dT\;/\;\overset{T_{\infty }}{\underset{T_{0}}{\int }}\left(dH/dT\right)/dT$$

where *T*_0_ and *T*_∞_ are the onset and end crystallization temperature, and *dH*/*dT* is the heat flow rate. In nonisothermal crystallization, the time scale t can be transformed from the temperature T by the equation:

(3) $$t=\left(T_{0}-T\right)/\emptyset$$

where ∅ is the cooling rate. Therefore, the graph of the relative degree of crystallinity *X****_t_*** versus time t can be plotted.

Avrami equation is generally applied to analyze the isothermal crystallization kinetics by the equation:

(4) $$X\left(t\right)=1-\exp\left(-kt^{n}\right)$$

where *X_t_* is the relative degree of crystallinity, t is crystallization time, n is the Avrami exponent, and k is a crystallization rate constant. n usually varies between 1 and 4 and can be influenced by the combined effect of nucleation and growth [42]. The double logarithmic form of this equation is:

(5) $$ln\left[-ln\left(1-X_{t}\right)\right]=nlnt+lnk$$

By plotting the graph of $ln\left[-ln\left(1-X_{t}\right)\right]$ vs. lnt, the values of *n* and *lnk* can be calculated by fitting the experimental data. Since the Avrami equation describes the isothermal crystallization process, Jeziorny suggested a method to modify the parameter *k* by introducing the cooling rate ∅ to describe the nonisothermal crystallization:

(6) $$\ln k_{c}\;=(\ln k)/\emptyset$$

The relative percentage crystallinity of *β* phase (*β*_c_) was calculated by the following equation:

(7) $$\beta_{c}=\frac{{\left(1-\lambda \right)}_{\beta }}{{\left(1-\lambda \right)}_{\alpha }+{\left(1-\lambda \right)}_{\beta }}$$

where (1 − λ) is the crystallinity of each phase and is calculated by Δ*H*/Δ*H_u_*. Δ*H* and Δ*H_u_* are the apparent and complete crystalline heats of fusion respectively. The value of Δ*H_u_* for 100% crystalline iPP is 209 J/g [43,44,45].

#### 2.3.5. Polarized Light Optical Microscopy (PLOM)

The crystalline morphologies of samples were investigated by PLOM (Eclipse LV100 POL, Nikon, Tokyo, Japan) coupled with a hot-stage (Linkam Scientific Instruments Ltd., Tad-worth, UK). A small piece of the sample was cut and placed between glass covers, melted at 200 °C [46] for 5 min, and cooled slowly to allow full crystallization. Then, the film samples were observed by PLOM.

## 3. Results and Discussions

### 3.1. Morphology and Structure of MXene

**Figure 2:**
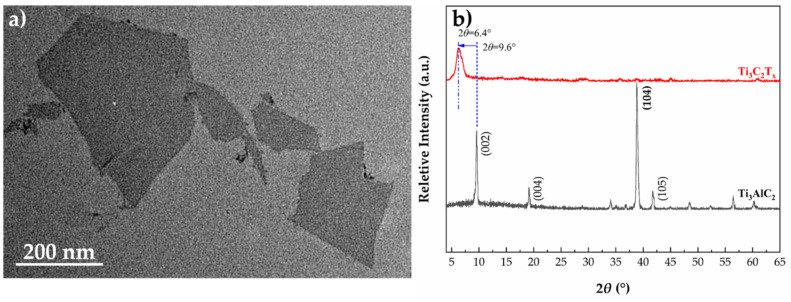
TEM image of Ti_3_C_2_T_x_ dispersed in distilled water (**a**) and XRD pattern of Ti_3_C_2_T_x_ powder (**b**).

As shown in Figure 2a, the MXene particles dispersed in distilled water contain flakes with lateral sizes around a few hundred nanometers. In addition, the X-ray spectra in Figure 2b indicates that a sharp (002) diffraction peak has shifted from 2*θ* ≈ 9.6° to 6.4° after etching, suggesting an expanded interlayer distance. The disappearance of the most intense (104) diffraction peak at 2*θ* ≈ 39°, which is representative of Ti_3_AlC_2_, further confirms the complete etching.

**Figure 3:**
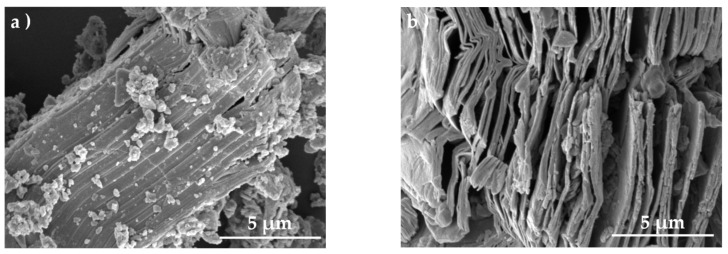
SEM images of (**a**) Ti**_3_**AlC**_2_** and (**b**) Ti_3_C_2_T_x_ before and after etching.

SEM was conducted to observe the morphologies of bulk Ti_3_AlC_2_ and Ti_3_C_2_T_x_. As shown in Figure 3a, the bulk MAX phase Ti_3_AlC_2_ exhibits a compact layered structure in which the flakes were closely stacked, and this particular structure can often be observed in ternary carbides [47]. After the selective etching process was completed, the flakes are weakly stacked and the interlayer distance increases. This morphology is also named accordion-like morphology. The expanded layered structure agrees well with the results of XRD and is possibly caused by escaped gas such as H_2_ during the etching process due to the exothermic reaction between HF and Al [48,49].

### 3.2. Nonisothermal Crystallization Behavior of β-iPP/MXene Composites

**Figure 4:**
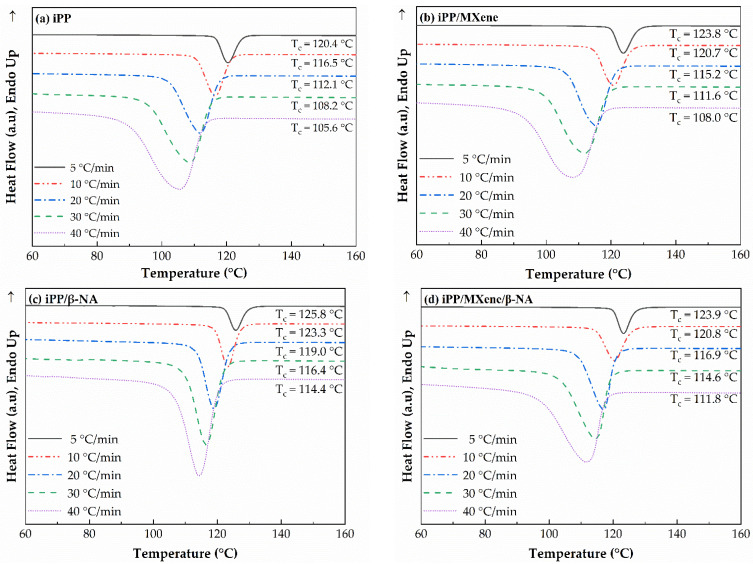
DSC cooling curves of (**a**) neat iPP, (**b**) iPP/MXene, (**c**) iPP/β-NA, and, (**d**) iPP/MXene/β-NA at cooling rates 5, 10, 20, 30, and 40 °C/min.

**Figure 5:**
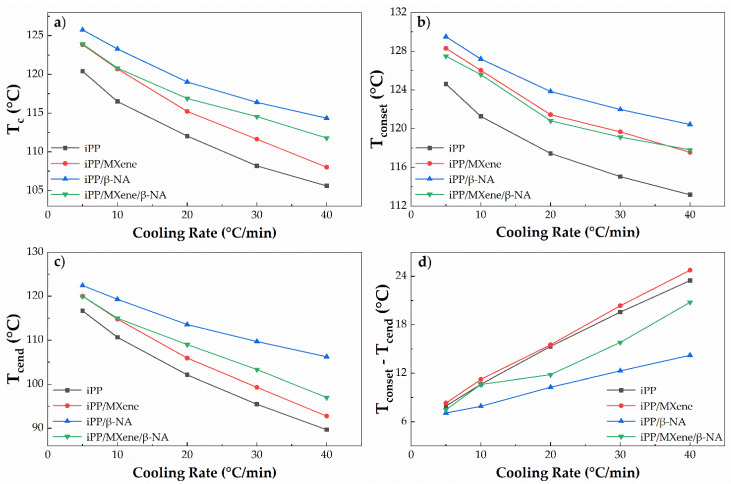
Plots of (**a**) T_c_, (**b**) T_conset_, (**c**) T_cend_, and (**d**) T_conset_—T_cend_ of four samples as a function of cooling rate at T_end_ = 50 °C.

The cooling curves of the four samples are plotted in Figure 4, and crystallization parameters including peak crystallization temperature (T_c_), onset and end crystallization temperatures (T_conset_, T_cend_), and crystallization peak width (T_conset_—T_cend_) are plotted in Figure 5. The larger the T_conset_—T_cend_, the greater the crystallization temperature range [43,50].

Firstly, it is found that for all samples, the lower the cooling rate is, the larger the value of T_c_, T_conset,_ and T_cend_. In other words, a lower cooling rate enables the sample to crystallize at a higher temperature. Moreover, the crystallization peak width T_conset_—T_cend_ decreases with a lower cooling rate, which indicates that the polymer chains get sufficient time to rearrange into crystalline phase. The variation trend for the four samples is slightly different. From Figure 5, it can be observed that the crystallization parameters T_c_, T_conset,_ and T_cend_ for neat iPP are the lowest, while the crystallization peak width is the largest. Neat iPP has the weakest crystallization ability among all samples, so it crystallizes at lower temperatures. When adding MXene, T_c_, T_conset_, and T_cend_ increase, but the crystallization peak width T_conset_—T_cend_ remains nearly unchanged, suggesting that the crystallization ability is improved at all cooling rates by adding MXene. The improved crystallization ability of iPP/MXene might be attributed to the heterogeneous nucleation effect of MXene [34]. When β-NA is added, T_c_, T_conset,_ and T_cend_ of iPP/β-NA are the highest while T_conset_—T_cend_ is the smallest, indicating that the addition of β-NA can greatly improve the crystallization ability of iPP, and the effect is more significant than the addition of MXene. This finding is consistent with the previous research results [51].

Interestingly, when MXene and β-NA are added simultaneously, the crystallization parameters of iPP/MXene/β-NA exhibit a different cooling rate dependence from other samples. At a lower cooling rate (≤10 °C/min), T_c_ and T_conset_—T_cend_ are both similar to iPP/MXene, indicating that under the combined effect of MXene and β-NA, the crystallization ability is not further improved. However, when the cooling rate increases (>10 °C/min), T_c_ and T_cend_ are higher than iPP/MXene but lower than iPP/β-NA, and the crystallization peak width is lower than iPP/MXene. This finding suggests that adding MXene and β-NA improve the crystallization temperature while narrowing the crystallization peak, and this effect is found more obvious under rapid cooling.

**Figure 6:**
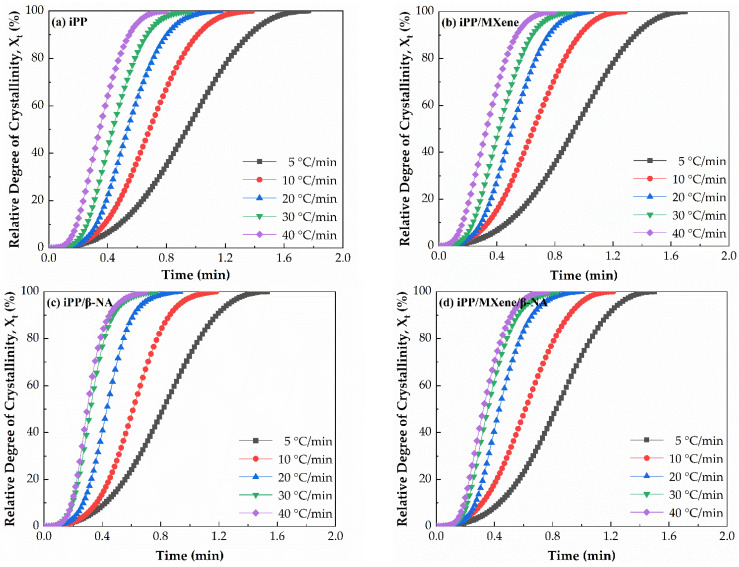
Plots of relative degree of crystallinity as a function of time of (**a**) iPP, (**b**) iPP/MXene, (**c**) iPP/β-NA, and (**d**) iPP/MXene/β-NA at different cooling rates.

**Figure 7:**
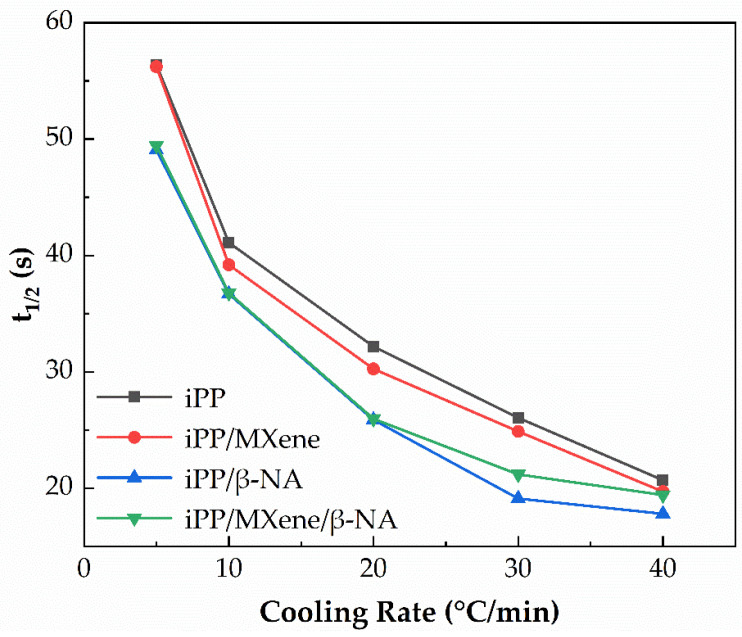
Half crystallization time t**_1/2_** of four samples at different cooling rates.

The cumulative relative degree of crystallinity (*X**_t_***) as a function of time curves at different cooling rates were plotted in Figure 6 from which the half crystallization time t_1/2_ is calculated when *X_t_* is 50%. The value of t_1/2_ can be used as a measure of crystallization rate and ability. In general, the larger the value of t_1/2_ is, the longer the time needed for the crystallization process. The plotted curves in Figure 6 and Figure 7 show that t_1/2_ of neat iPP is higher than the other three composites at all cooling rates, suggesting its crystallization rate is the lowest. t_1/2_ of iPP/MXene/β-NA at lower cooling rates is almost the same as that of iPP/β-NA, while increases significantly at higher cooling rates, proving that the crystallization ability of iPP/MXene/β-NA is not as good as iPP/β-NA. This result agrees with findings from DSC cooling curves.

The results from the Avrami method are reported in Table 1, where the value of *n* strongly depends on the mechanism of the crystal growth and *k* can be used as a measure of crystallization rate. *n* for samples at all cooling rates varies between 2.5 and 3.2. The values can be attributed to heterogeneous nucleation and three-dimensional growth in crystallites [52,53], suggesting that the addition of the above fillers does not alter the crystallization mechanism of iPP. In addition, it can be seen that the value of *lnk* increases after the addition of MXene and β-NA, indicating that the crystallization rate is improved. For iPP/MXene/β-NA, the value of *lnk* is higher than iPP/MXene, but lower than iPP/β-NA, and this variation trend is the same as t_1/2_.

**Figure 8:**
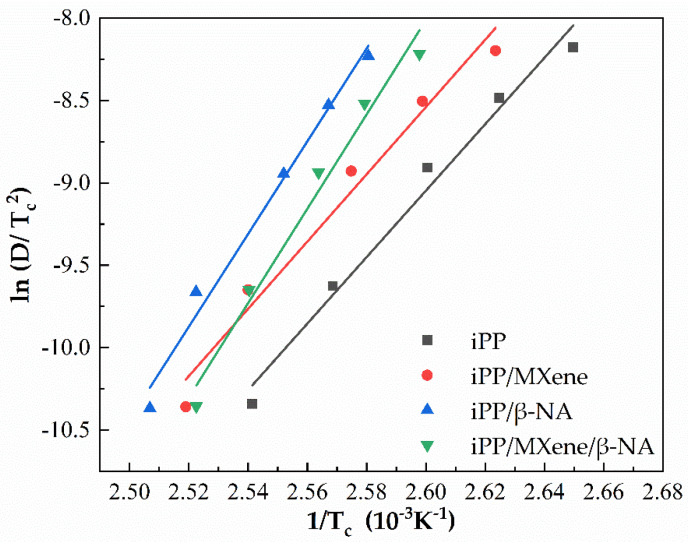
Plots of ln(D/T_c_^2^) versus 1/T**_c_** of four samples to calculate nonisothermal activation energy *E_c_* by Kissinger method.

The nonisothermal crystallization activation energy *E****_c_*** represents the energy barrier of crystallization. In general, the larger the *E_c_* is, the more difficult for the occurrence of the crystallization process [54,55]. The Kissinger plot of studied samples is shown in Figure 8, the slope can be used to calculate the activation energy of the sample. Table 1 revealed that after the addition of MXene and β-NA, the crystallization energy barrier is lowered and the crystallization becomes easier. However, when MXene and β-NA are added simultaneously, the energy barrier increases again suggesting that the crystallization process becomes more difficult. Combining the other crystallization parameters and previous study [51], the results suggest that there exists a competitive relationship between MXene and β-NA as fillers.

### 3.3. Melting and Polymorphic Behavior of β-iPP/MXene Composites

**Figure 9:**
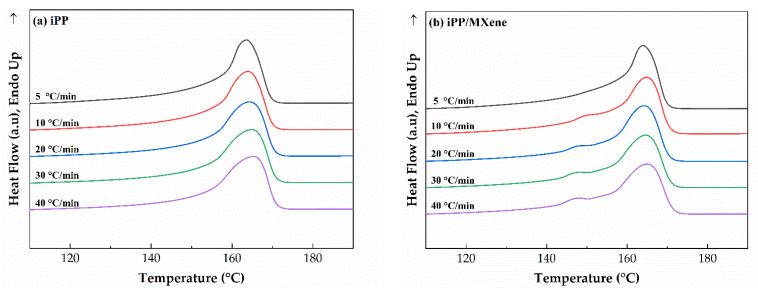

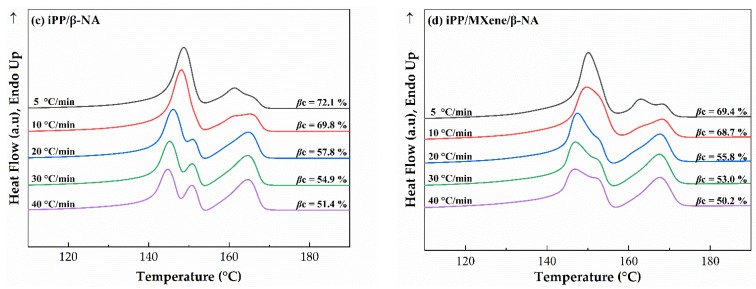
DSC melting curves of (**a**) neat iPP, (**b**) iPP/MXene, (**c**) iPP/β-NA, and (**d**) iPP/MXene/β-NA at cooling rates 5, 10, 20, 30, and 40 °C/min.

The subsequent melting curves at a rate of 10 °C/min of four samples are shown in Figure 9. It can be observed that both iPP and iPP/MXene show only one melting peak between 160–170 °C, therefore the addition of MXene promotes the crystallization of α-crystals. At the same time, when β-NA is added, iPP/β-NA and iPP/MXene/β-NA show β-crystals melting peaks between 140–155 °C. The melting behaviors of iPP/β-NA and iPP/MXene/β-NA show different dependence on cooling rates. For iPP/β-NA, as the cooling rate increases, the β peak gradually splits into two independent peaks, proving the formation of β-crystals with different crystalline perfections. On the contrary, this phenomenon is found weaker in iPP/MXene/β-NA, suggesting that the melting behavior of iPP/MXene/β-NA might be less dependent on the cooling rates.

**Figure 10:**
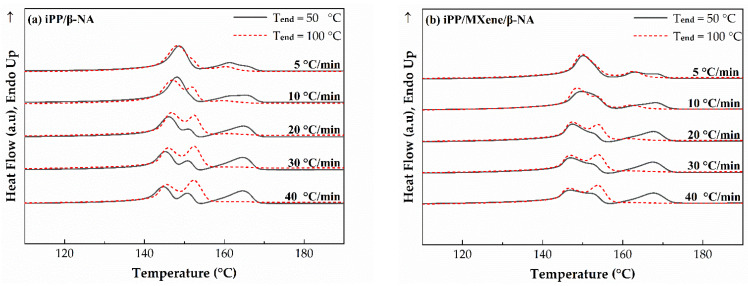
DSC melting curves of (**a**) iPP/β-NA and (**b**) iPP/MXene/β-NA at two end temperatures.

**Figure 11:**
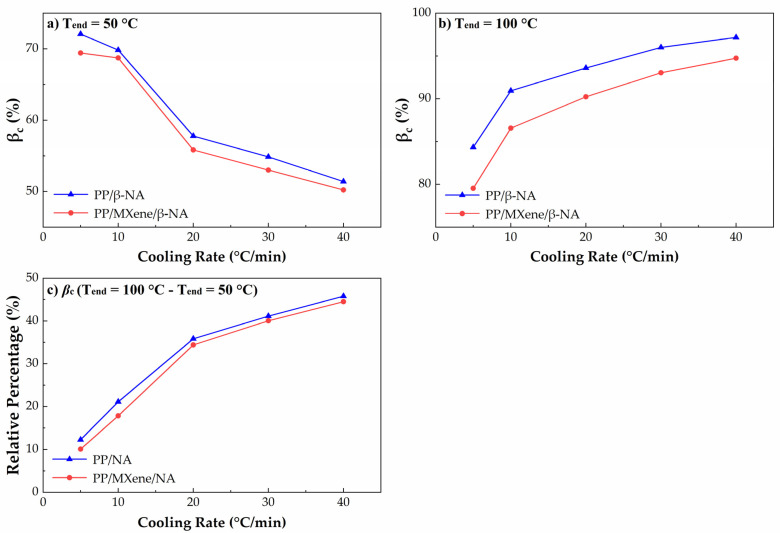
Plots of relative percentage crystallinity of β phase at (**a**) T_end_ = 50 °C, (**b**) T_end_ = 100 °C, and (**c**) their differences *β*_c_ (T_end_ = 100 °C—T_end_ = 50 °C) at 5, 10, 20, 30, and 40 °C/min.

The relative percentage crystallinity of β-phase (*β*_c_) is calculated by Equation (7) and shown in Figure 11. Figure 11a shows that at T_end_ = 50 °C, *β*_c_ gradually decreases with increasing cooling rate, which seems that a lower cooling rate is more beneficial for the formation of high content β-crystals. However, the *β*_c_ calculated from DSC is affected by two factors. One is the ability to form β-crystals, i.e., the amount of β-crystals. The second factor is the thermal stability of β-crystals. According to the study of Varga et al. [56,57,58], during the partial melting of β-crystals, β-iPP cooled below a critical temperature (T_end_ = 100–105 °C) would recrystallize into α-iPP in the subsequent melting behavior. The exothermic recrystallization peak of β-crystals coincides with the endothermic melting peak, which would interfere with the accuracy of *β*_c_ on DSC curves. To comparatively study the polymorphic behavior at different cooling rates, we compared the melting curves at two end crystallization temperatures T_end_ = 50 °C and T_end_ = 100 °C, the melting curves and calculated *β*_c_ are plotted in Figure 10 and Figure 11.

At T_end_ = 50 °C, it is found in Figure 11a that when the cooling rate decreases, the β-crystals content would increase. To eliminate the effects of thermal stability of β-crystals, T_end_ is set to 100 °C so that no β-α recrystallization would occur. At T_end_ = 100 °C, the β-crystals melting peaks in Figure 10 are wider and larger, suggesting that the β-crystal content is also higher. The possible reason behind is at T_end_ = 100 °C the interference of recrystallization is eliminated, so the β-crystals content is much higher. Secondly, Figure 11b indicates that *β***_c_** increases with higher cooling rates. After β-α recrystallization interference is removed, rapid cooling favors the formation of more β-crystals. When MXene is added, β crystallization is inhibited due to the competitive effect between MXene and β-NA, while the cooling rate dependence remains unchanged. Figure 11c shows the *β*_c_ difference at T_end_ = 100 °C and T_end_ = 50 °C, when cooling rate increases, the difference between *β*_c_ is higher. The possible explanation is proposed as the following, when the cooling rate increases more thermally unstable β-crystals tend to participate in β-α recrystallization. Moreover, when MXene is added, *β*_c_ decreases indicating that the addition of MXene can decrease the content of β-crystals with low thermal stability. From melting curves, it can be summarized that the crystallization behavior and polymorphic behavior of MXene/β-iPP composites can be influenced by cooling rates and thermal conditions.

### 3.4. Effects of Thermal Conditions

In order to comprehensively understand the influences of thermal conditions on crystallization and polymorphic behavior of the composites, three groups of samples with different thermal histories were molded and named Fast Cooling Rate, Medium Cooling Rate, and Slow Cooling Rate.

#### 3.4.1. PLOM Observation

**Figure 12:**
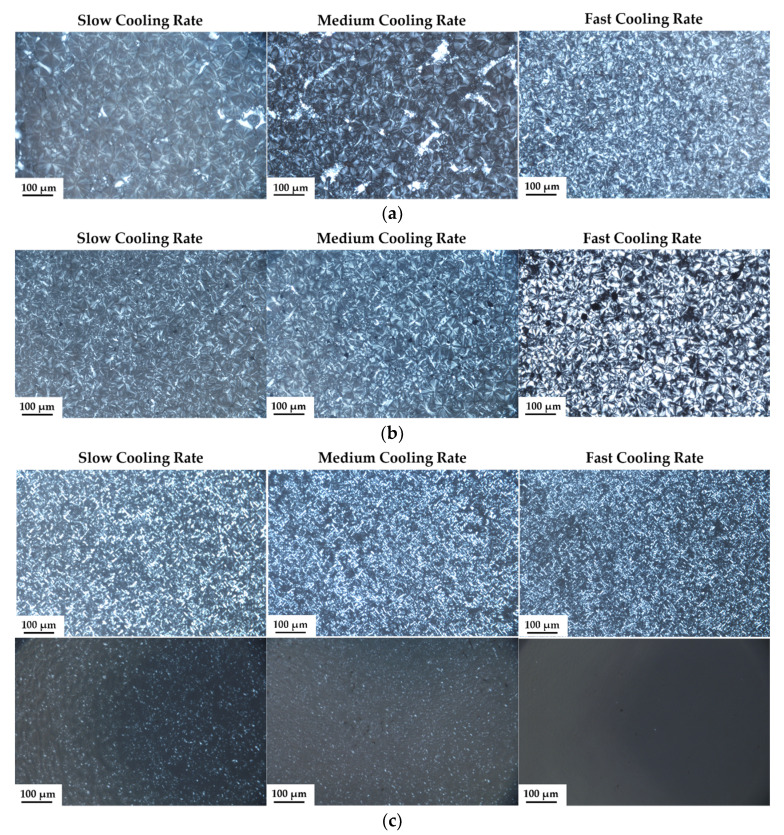

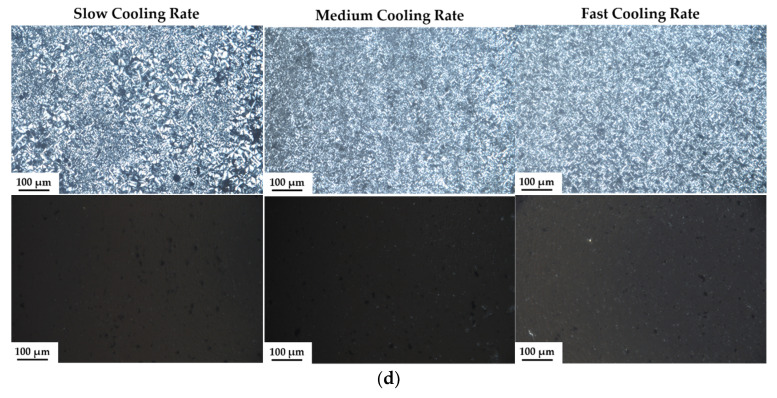
PLOM images of (**a**) iPP, (**b**) iPP/MXene, (**c**) iPP/β-NA, and (**d**) iPP/MXene/β-NA with different thermal histories. iPP/β-NA and iPP/MXene/β-NA were remelted to 165 °C to observe the residual morphologies. The scale bar represents 100 μm.

The crystallization morphology of the samples was observed by PLOM and the photos are shown in Figure 12. It is found that iPP and iPP/MXene exhibit spherulites morphology that corresponds to the α-crystals. When the cooling rate increases, crystal nucleation is more favorable while crystal growth is confined. As a result, it is observed that the spherulite size decreases gradually with increasing cooling rates.

Figure 12c,d indicate that after addition of β-NA, a large number of β-crystals appear. In iPP/β-NA, the β-crystals dominate, and crystal size decreases gradually with increasing cooling rates. At slow cooling rate, iPP/MXene/β-NA exhibits obvious and well-grown α-crystals, which decrease in size as the cooling rate increases. The PLOM results agree well with the previous findings.

#### 3.4.2. DSC Analysis

**Figure 13:**
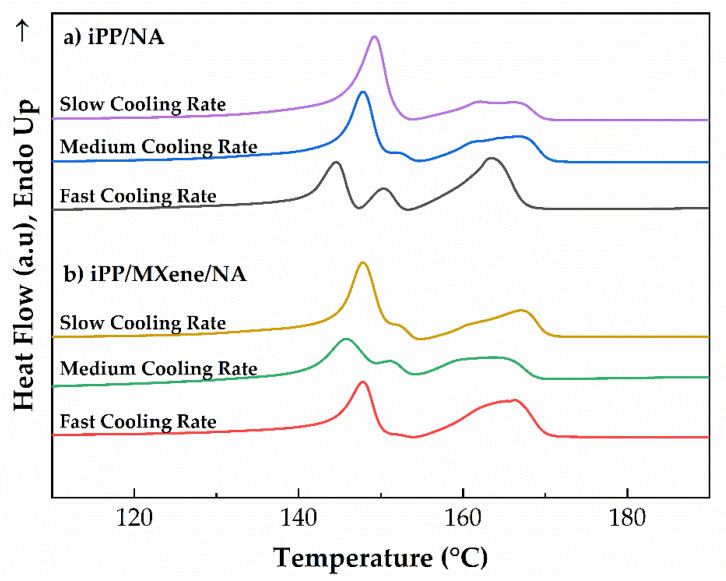
DSC melting curves of (**a**) iPP/β-NA and (**b**) iPP/MXene/β-NA of different thermal histories.

**Figure 14:**
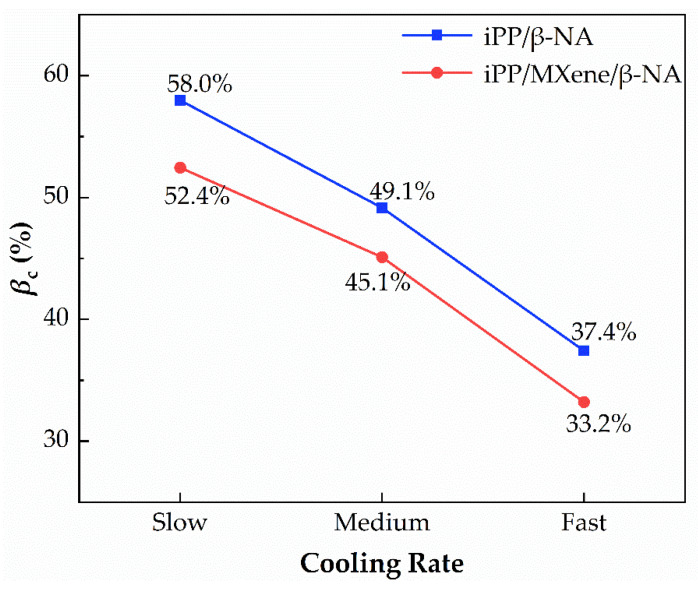
*β***_c_** of iPP/β-NA and iPP/MXene/β-NA of different thermal histories.

To analyze the polymorphic behavior, the melting curves of iPP/β-NA and iPP/MXene/β-NA at a cooling rate of 10 °C/min are shown in Figure 13 and the *β***_c_** calculated from the curves as a function of cooling rate is plotted in Figure 14. It is found that as the cooling rate increases, the β-crystals peak becomes weaker and *β*_c_ decreases accordingly. Furthermore, the β-crystal content in iPP/β-NA is higher than iPP/MXene/β-NA at all cooling rates. This variation trend in *β*_c_ as a function of cooling rate is consistent with the results from nonisothermal crystallization analysis in the previous part.

#### 3.4.3. X-ray Diffraction (XRD)

**Figure 15:**
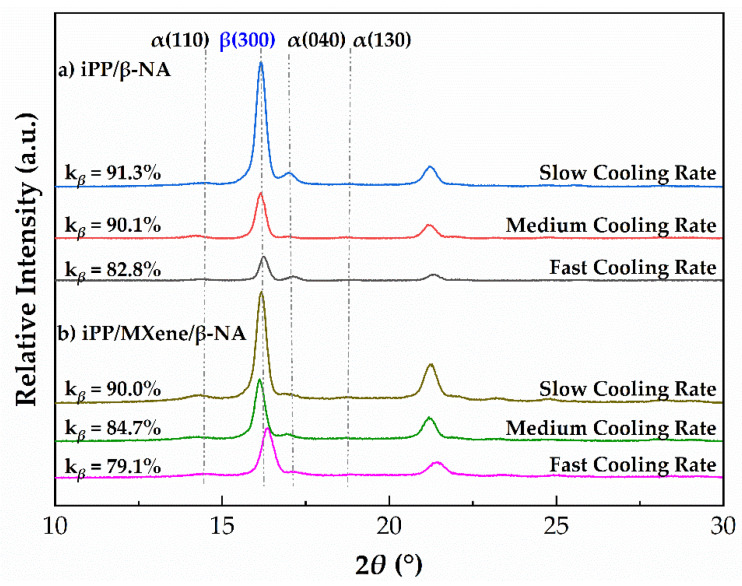
XRD pattern of (**a**) iPP/β-NA and (**b**) iPP/MXene/β-NA with different thermal histories.

The XRD results of iPP/β-NA and iPP/MXene/β-NA were further compared in Figure 15. From the XRD patterns, four main peaks can be found at 2*θ* ≈ 14.1°, 16.1°, 16.9°, and 18.6° that correspond to α (110), β (300), α (040), and α (130) diffractions [45,59,60]. Since XRD is performed at room temperature, during which the effects of β-α recrystallization can be excluded. Therefore, the k*_β_* value calculated from XRD patterns at all cooling rates is higher than *β*_c_ value calculated from DSC melting curves. Apart from that, when the cooling rate increases, the k*_β_* decreases from 91.3 to 82.8% in iPP/β-NA and from 90.0 to 79.1 % in iPP/MXene/β-NA. This trend also agrees with findings from DSC melting curves.

## 4. Conclusions

The effects of MXene on nonisothermal crystallization behavior β-iPP/MXene composites were investigated. The DSC analysis shows that the crystallization ability of the samples follows the order iPP/β-NA, iPP/MXene/β-NA, iPP/MXene, iPP. When MXene and β-NA are added separately, the crystallization temperature of composites increases with lower cooling rates. However, when MXene and β-NA are added simultaneously, they tend to compete with each other. Due to the low thermal stability of β-crystals, the melting behaviors at two end temperatures T_end_ = 50 °C and T_end_ = 100 °C show different variation tendency. When the crystallization end temperature T_end_ = 50 °C, a lower cooling rate leads to more formation β-crystals. But this trend is reversed at T_end_ = 100 °C because more unstable β-crystals would participate in β-α recrystallization with higher cooling rates. Moreover, the experimental phenomenon that k*_β_* calculated from XRD is higher than *β*_c_ calculated from DSC at all cooling rates further proves the β-α recrystallization. The study paves a method to prepare β-iPP/MXene composites with adjustable crystallization and polymorphic behavior, which can provide new insight to develop high performance iPP/MXene composites with excellent mechanical properties in future work.

## Figures and Tables

**Table 1:** Crystallization kinetics parameters of four samples at different cooling rates. *E_c_* represents the crystallization activation energy calculated by Kissinger plot. t*_1/2_* represents the half crystallization time calculated from the relative degree of crystallinity vs. time graph. *n* and *lnk* represent the Avrami exponent and crystallization rate constant calculated from Avrami and Jeziorny method.

Sample	*E_c_* (kJ/mol)	Cooling Rate (°/min)	t_1/2_ (s)	*N*	*lnk*
iPP	−167.5	5	56.4	2.6	−0.09
		10	41.1	2.8	0.72
		20	32.2	3.2	1.45
		30	26.1	3.2	2.14
		40	20.7	3.1	2.66
iPP/MXene	−185.5	5	56.2	2.5	−0.08
		10	39.2	2.6	0.84
		20	30.3	3.0	1.59
		30	24.9	3.0	2.13
		40	19.7	2.7	2.57
iPP/β-NA	−233.8	5	49.1	2.6	0.27
		10	36.7	2.9	1.07
		20	25.9	3.1	2.12
		30	19.1	3.1	2.86
		40	17.8	3.3	3.25
iPP/MXene/β-NA	−218.1	5	49.4	2.6	0.24
		10	36.8	2.6	1.00
		20	26.0	3.0	1.95
		30	21.2	3.1	2.48
		40	19.4	3.2	3.01

## Data Availability

Not applicable.

## References

[1] Natta G., Corradini P. (1960). Structure and properties of isotactic polypropylene. Nuovo Cim. Suppl..

[2] Li Y., Liu H., Huang X., Song X., Kang J., Chen Z., Zeng F., Chen J. (2020). Investigation on the Roles of β-Nucleating Agents in Crystallization and Polymorphic Behavior of Isotactic Polypropylene. Polym. Sci. Ser. A.

[3] Sun J., Li Q., Yao X.-J., Hu J.-S., Qi Y. (2013). Influence of two liquid crystalline polysiloxanes with different average molecular weight as new β-nucleator on crystallization structure of isotactic polypropylene. Polym. Bull..

[4] Bruckner S., Meille S.V., Petraccone V., Pirozzi B. (1991). Polymorphism in isotactic polypropylene. Prog. Polym. Sci..

[5] Padden F.J., Keith H.D. (1959). Spherulitic Crystallization in Polypropylene. J. Appl. Phys..

[6] Kang J., Li J., Chen S., Zhu S., Li H., Cao Y., Yang F., Xiang M. (2013). Hydrogenated petroleum resin effect on the crystallization of isotactic polypropylene. J. Appl. Polym. Sci..

[7] Jiang X., Fang Y., Yu Y., Kang J., Cao Y., Xiang M., Li L., Sheng X., Hao Z. (2019). Exploring the Effects of Stereo-Defect Distribution on Nonisothermal Crystallization and Melting Behavior of beta-Nucleated Isotactic Polypropylene/Graphene Oxide Composites. ACS Omega.

[8] Pawlak A., Piorkowska E. (2001). Crystallization of isotactic polypropylene in a temperature gradient. Colloid Polym. Sci..

[9] Zhang B., Chen J., Ji F., Zhang X., Zheng G., Shen C. (2012). Effects of melt structure on shear-induced β-cylindrites of isotactic polypropylene. Polymer.

[10] Koscher E., Fulchiron R. (2002). Influence of shear on polypropylene crystallization: Morphology development and kinetics. Polymer.

[11] Grein C. (2005). Toughness of Neat, Rubber Modified and Filled β-nucleated Polypropylene: From Fundamentals to Applications. Intrinsic Molecular Mobility and Toughness of Polymers II.

[12] Wang S.-W., Yang W., Bao R.-Y., Wang B., Xie B.-H., Yang M.-B. (2010). The enhanced nucleating ability of carbon nanotube-supported β-nucleating agent in isotactic polypropylene. Colloid Polym. Sci..

[13] Yu Y., Zeng F., Chen J., Jian K., Ming X. (2018). Regulating polycrystalline behavior of the β-nucleated isotactic polypropylene/graphene oxide composites by melt memory effect. Polym. Compos..

[14] Yu Y., Xu R., Chen J., Kang J., Xiang M., Li Y., Li L., Sheng X. (2019). Ordered structure effects on β-nucleated isotactic polypropylene/graphene oxide composites with different thermal histories. RSC Adv..

[15] Ahmadivand A., Gerislioglu B., Ramezani Z. (2019). Gated graphene island-enabled tunable charge transfer plasmon terahertz metamodulator. Nanoscale.

[16] Yadav A., Gerislioglu B., Ahmadivand A., Kaushik A., Cheng G.J., Ouyang Z., Wang Q., Yadav V.S., Mishra Y.K., Wu Y. (2021). Controlled self-assembly of plasmon-based photonic nanocrystals for high performance photonic technologies. Nano Today.

[17] Naguib M., Kurtoglu M., Presser V., Lu J., Niu J., Heon M., Hultman L., Gogotsi Y., Barsoum M.W. (2011). Two-dimensional nanocrystals produced by exfoliation of Ti_3_AlC_2_. Adv. Mater..

[18] Verger L., Natu V., Carey M., Barsoum M.W. (2019). MXenes: An Introduction of Their Synthesis, Select Properties, and Applications. Trends Chem..

[19] Jimmy J., Kandasubramanian B. (2020). Mxene functionalized polymer composites: Synthesis and applications. Eur. Polym. J..

[20] Barsoum M.W. (2013). MAX Phases: Properties of Machinable Ternary Carbides and Nitrides.

[21] Zhang H. (2015). Ultrathin Two-Dimensional Nanomaterials. Acs Nano.

[22] Verger L., Xu C., Natu V., Cheng H.-M., Ren W., Barsoum M.W. (2019). Overview of the synthesis of MXenes and other ultrathin 2D transition metal carbides and nitrides. Curr. Opin. Solid State Mater. Sci..

[23] Hu M., Hu T., Li Z., Yang Y., Cheng R., Yang J., Cui C., Wang X. (2018). Surface Functional Groups and Interlayer Water Determine the Electrochemical Capacitance of Ti_3_C_2_ T x MXene. ACS Nano.

[24] Jiang Q., Lei Y., Liang H., Xi K., Alshareef H.N. (2020). Review of MXene Electrochemical Microsupercapacitors. Energy Storage Mater..

[25] Aslam M.K., Xu M. (2020). A Mini-Review: MXene composites for sodium/potassium-ion batteries. Nanoscale.

[26] Mathis T.S., Maleski K., Goad A., Sarycheva A., Anayee M., Foucher A.C., Hantanasirisakul K., Shuck C.E., Stach E.A., Gogotsi Y. (2021). Modified MAX Phase Synthesis for Environmentally Stable and Highly Conductive Ti_3_C_2_ MXene. ACS Nano.

[27] Garg R., Agarwal A., Agarwal M. (2020). A review on MXene for energy storage application: Effect of interlayer distance. Mater. Res. Express.

[28] Lukatskaya M.R., Mashtalir O., Ren C.E., Dall’Agnese Y., Rozier P., Taberna P.L., Naguib M., Simon P., Barsoum M.W., Gogotsi Y. (2013). Cation intercalation and high volumetric capacitance of two-dimensional titanium carbide. Science.

[29] Ling Z., Ren C.E., Zhao M.Q., Yang J., Giammarco J.M., Qiu J., Barsoum M.W., Gogotsi Y. (2014). Flexible and conductive MXene films and nanocomposites with high capacitance. Proc. Natl. Acad. Sci. USA.

[30] Yi Z., Yang J., Liu X., Mao L., Cui L., Liu Y. (2019). Enhanced mechanical properties of poly(lactic acid) composites with ultrathin nanosheets of MXene modified by stearic acid. J. Appl. Polym. Sci..

[31] Zhang H., Wang L., Chen Q., Li P., Zhou A., Cao X., Hu Q. (2016). Preparation, mechanical and anti-friction performance of MXene/polymer composites. Mater. Des..

[32] Wan Y.-J., Li X.-M., Zhu P.-L., Sun R., Wong C.-P., Liao W.-H. (2020). Lightweight, flexible MXene/polymer film with simultaneously excellent mechanical property and high-performance electromagnetic interference shielding. Compos. Part A Appl. Sci. Manuf..

[33] Huang Z., Wang S., Kota S., Pan Q., Barsoum M.W., Li C.Y. (2016). Structure and crystallization behavior of poly(ethylene oxide)/Ti3C2Tx MXene nanocomposites. Polymer.

[34] Peng W., Hu R., Jiang W., Kang J., Xiang M. (2021). Effects of MXene on Nonisothermal Crystallization Kinetics of Isotactic Polypropylene. ACS Omega.

[35] Kang J., Weng G., Chen Z., Chen J., Cao Y., Yang F., Xiang M. (2014). New understanding in the influence of melt structure and β-nucleating agents on the polymorphic behavior of isotactic polypropylene. RSC Adv..

[36] Luo F., Geng C., Wang K., Deng H., Chen F., Fu Q., Na B. (2009). New Understanding in Tuning Toughness of β-Polypropylene: The Role of β-Nucleated Crystalline Morphology. Macromolecules.

[37] Yu Y., Zeng F., Chen J., Kang J., Yang F., Cao Y., Xiang M. (2018). Isothermal crystallization kinetics and subsequent melting behavior of β-nucleated isotactic polypropylene/graphene oxide composites with different ordered structure. Polym. Int..

[38] Yu Y., Jiang X., Fang Y., Chen J., Kang J., Cao Y., Xiang M. (2019). Investigation on the Effect of Hyperbranched Polyester Grafted Graphene Oxide on the Crystallization Behaviors of beta-Nucleated Isotactic Polypropylene. Polymers..

[39] Jones A.T., Aizlewood J.M., Beckett D.R. (1964). Crystalline Forms of Isotactic Polypropylene. Macromol. Chem. Phys..

[40] Cao X., Wu M., Zhou A., Wang Y., He X., Wang L. (2017). Non-isothermal crystallization and thermal degradation kinetics of MXene/linear low-density polyethylene nanocomposites. e-Polymers.

[41] Layachi A., Makhlouf A., Frihi D., Satha H., Belaadi A., Seguela R. (2019). Non-isothermal crystallization kinetics and nucleation behavior of isotactic polypropylene composites with micro-talc. J. Therm. Anal. Calorim..

[42] Avrami M. (1939). Kinetics of Phase Change. I General Theory. J. Chem. Phys..

[43] Kang J., Wang B., Peng H., Li J., Chen J., Gai J., Cao Y., Li H., Yang F., Xiang M. (2014). Investigation on the dynamic crystallization and melting behavior of β-nucleated isotactic polypropylene with different stereo-defect distribution-the role of dual-selective β-nucleation agent. Polym. Adv. Technol..

[44] Li J., Cheung W., Jia D. (1999). A study on the heat of fusion of β-polypropylene. Polymer.

[45] Marco C., Gómez M.A., Ellis G., Arribas J.M. (2002). Activity of a β-nucleating agent for isotactic polypropylene and its influence on polymorphic transitions. J. Appl. Polym. Sci..

[46] Zhang F., Jiang W., Song X., Kang J., Xiang M. (2021). Effects of Hyperbranched Polyester-Modified Carbon Nanotubes on the Crystallization Kinetics of Polylactic Acid. ACS Omega.

[47] Alhabeb M., Maleski K., Anasori B., Lelyukh P., Clark L., Sin S., Gogotsi Y. (2017). Guidelines for Synthesis and Processing of Two-Dimensional Titanium Carbide (Ti_3_C_2_Tx MXene). Chem. Mater..

[48] Li C., Zhang X., Wang K., Sun X., Ma Y. (2020). Accordion-like titanium carbide (MXene) with high crystallinity as fast intercalative anode for high-rate lithium-ion capacitors. Chin. Chem. Lett..

[49] Ab Alim N.N.N., Mohamed Saheed M.S., Mohamed N.M., Mohamed Saheed M.S. (2019). Highly flexible and stretchable 3D graphene/MXene composite thin film. Mater. Today Proc..

[50] Gao J., Cao X., Zhang C., Hu W. (2013). Non-isothermal crystallization kinetics of polypropylene/MAP-POSS nanocomposites. Polym. Bull..

[51] Peng W., Kang J., Song X., Zhang Y., Hu B., Cao Y., Xiang M. (2021). Investigation on the Effects of MXene and β-Nucleating Agent on the Crystallization Behavior of Isotactic Polypropylene. Polymers.

[52] Manchado M., Blagiotti J., Torre L., Kenny J.M. (2010). Effects of reinforcing fibers on the crystallization of polypropylene. Polym. Eng. Sci..

[53] Eder M., Wlochowicz A. (1983). Kinetics of non-isothermal crystallization of polyethylene and polypropylene. Polymer.

[54] Kang J., He J., Chen Z., Yang F., Chen J., Cao Y., Xiang M. (2015). Effects of β-nucleating agent and crystallization conditions on the crystallization behavior and polymorphic composition of isotactic polypropylene/multi-walled carbon nanotubes composites. Polym. Adv. Technol..

[55] Papageorgiou G.Z., Achilias D.S., Bikiaris D.N., Karayannidis G.P. (2005). Crystallization kinetics and nucleation activity of filler in polypropylene/surface-treated SiO2 nanocomposites. Thermochim. Acta.

[56] Varga J. (1986). Melting memory effect of the β-modification of polypropylene. J. Therm. Anal..

[57] Horvath Z., Sajo I.E., Stoll K., Menyhard A., Varga J. (2010). The effect of molecular mass on the polymorphism and crystalline structure of isotactic polypropylene. eXPRESS Polym. Lett..

[58] Varga J. (2007). β-Modification of Isotactic Polypropylene: Preparation, Structure, Processing, Properties, and Application. J. Macromol. Sci. Part B.

[59] Varga J., And I.M., Ehrenstein G.W. (1999). Highly active thermally stable β-nucleating agents for isotactic polypropylene. J. Appl. Polym. Sci..

[60] Zhang Q., Peng H., Kang J., Cao Y., Xiang M. (2017). Effects of melt structure on non-isothermal crystallization behavior of isotactic polypropylene nucleated with α/β compounded nucleating agents. Polym. Eng. Sci..

